# Digging Deeper: Advancements in Visualization of Inhibitory Synapses in Neurodegenerative Disorders

**DOI:** 10.3390/ijms222212470

**Published:** 2021-11-18

**Authors:** Snježana Radulović, Sowmya Sunkara, Christa Maurer, Gerd Leitinger

**Affiliations:** 1 Gottfried Schatz Research Center, Division of Cell Biology, Histology and Embryology, Medical University of Graz, 8010 Graz, Austria; snjezana.radulovic@medunigraz.at (S.R.); sowmya.sunkara@medunigraz.at (S.S.); 2 Gottfried Schatz Research Center, Division of Macroscopic and Clinical Anatomy, Medical University of Graz, 8010 Graz, Austria; christa.maurer@medunigraz.at

**Keywords:** neurodegeneration, inhibitory synapse, synaptic plasticity, Alzheimer’s disease, EM, STORM, STED, SIM

## Abstract

Recent research has provided strong evidence that neurodegeneration may develop from an imbalance between synaptic structural components in the brain. Lately, inhibitory synapses communicating via the neurotransmitters GABA or glycine have come to the center of attention. Increasing evidence suggests that imbalance in the structural composition of inhibitory synapses affect deeply the ability of neurons to communicate effectively over synaptic connections. Progressive failure of synaptic plasticity and memory are thus hallmarks of neurodegenerative diseases. In order to prove that structural changes at synapses contribute to neurodegeneration, we need to visualize single-molecule interactions at synaptic sites in an exact spatial and time frame. This visualization has been restricted in terms of spatial and temporal resolution. New developments in electron microscopy and super-resolution microscopy have improved spatial and time resolution tremendously, opening up numerous possibilities. Here we critically review current and recently developed methods for high-resolution visualization of inhibitory synapses in the context of neurodegenerative diseases. We present advantages, strengths, weaknesses, and current limitations for selected methods in research, as well as present a future perspective. A range of new options has become available that will soon help understand the involvement of inhibitory synapses in neurodegenerative disorders.

## 1. Introduction

Neurodegenerative diseases (ND) are a heterogeneous group of disorders that primarily damage the structure and function of neurons, thereby causing synaptic dysfunction. The presence of a range of clinical symptoms along with characteristic protein aggregates defines major NDs, such as Alzheimer’s disease (AD), Parkinson’s disease, Huntington’s disease, multiple sclerosis, and amyotrophic lateral sclerosis. The clinical symptoms of patients with ND include but are not limited to: memory loss (dementia); movement and cognitive impairment; disorientation; and changes in personality characteristics that ultimately harm life expectancy and quality of life [1]. Strong research evidence correlates the majority of these clinical symptoms with slippage in synaptic plasticity in ND [2,3]. Synaptic plasticity refers to the structural changes that occur at pre and postsynaptic sites to facilitate effective communication between two new or existing neurons [4]. Recent studies reveal the involvement of plasticity of inhibitory synapses (GABAergic and glycinergic) in synaptic dysfunction and disease progression in ND [5]. Evolving methods of visualization have enabled researchers to contribute greatly to understanding synaptic plasticity in NDs. Hence, in our review, we focus mainly on inhibitory synaptic plasticity in ND, with emphasis on Alzheimer’s disease, and discuss the established and evolving methods of visualization that have contributed to our knowledge of changes in inhibitory synapse plasticity so far.

**Figure 1:**
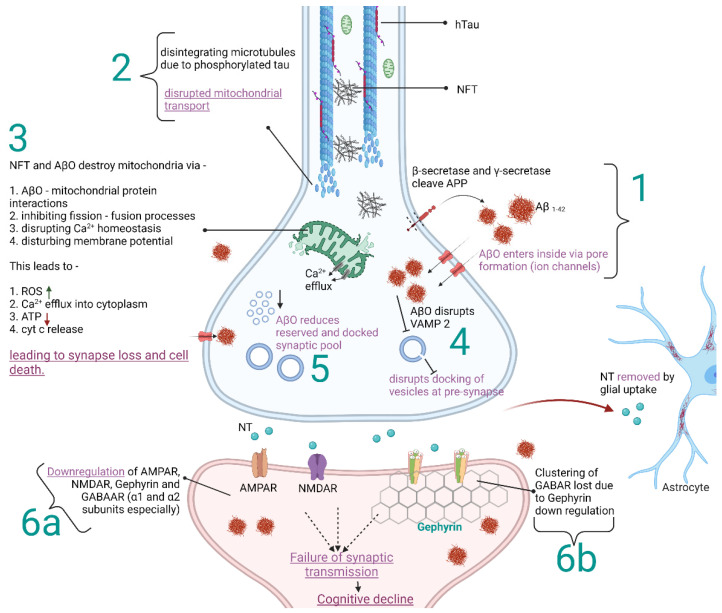
Schematic representation of the direct and indirect influence of amyloid-beta oligomers (AβO), hyperphosphorylated tau (hTau), and neurofibrillary tangles (NFT) on synaptic components in the context of Alzheimer’s disease (AD). The figure represents an example of the influence of protein aggregates and biomarkers on synaptic plasticity at functional and molecular levels in neurodegenerative diseases, which affects the cognitive functions of the brain. α-amino-3-hydroxy-5-methyl-4-isoxazole propionic acid receptor (AMPAR) and N-methyl D-aspartate Receptors (NMDAR) are glutamate receptors present on the postsynaptic membrane. GABAAR—γ-aminobutyric acid type-B receptors; APP—amyloid-beta precursor protein; Ca^2+^—calcium ion; ROS—reactive oxygen species; ATP—adenosine triphosphate; cyt c—cytochrome c; VAMP 2—Vesicle-associated membrane protein 2; NT—neurotransmitter [3,10,11,12,13,14,15,16,17,18,19,20,21].

Alzheimer’s disease, similar to the majority of NDs, is either familial or sporadic, or both combined, but there is no defined start point for the origin of the disease. AD is pathologically defined by the presence of aggregated amyloid-beta oligomers (AβOs, peptides of 36–43 amino acids), aggregated tau protein, neurodegeneration, and neuronal injury [6,7]. Figure 1 shows a schematic representation of the effects of AβOs, tau phosphorylation, and neurofibrillary tangles (NFT) on synaptic plasticity. Pathomechanics of AD are shown as an example to illustrate the overview of protein aggregates and biomarkers involved in ND and its direct and indirect role in inhibitory synaptic plasticity. Often, there is a long asymptomatic phase before the onset of the early stages of the disease. Inhibitory neurons are important to maintain synaptic balance for healthy neuronal functioning in the brain. In the adult brain, GABAergic neurons are the major inhibitory neurons that are critical to maintaining synaptic balance [5]. Any loophole in inhibitory synaptic plasticity can trigger accidents in neuronal communication, leading to progressive neurodegenerative diseases [8,9].

AD is characterized by memory loss and cognitive impairment, which is linked to the loss of inhibitory synapses in the brain [22]. Due to the post-mitotic character of most neurons in the brain, the ND is irreversible. Today there is no approved cure for ND. The current treatment strategy is to implement drugs that temporarily halt the symptoms and progression of ND (see Table 1 for an overview). However, several pathways and mechanisms are emerging that may become the basis for new drugs in the future. In recent years, accumulating evidence from various novel visualization techniques has begun to confirm the involvement of Aβ oligomers and tau protein in the loss of inhibitory neurons’ plasticity [3,5,19,20,21,23,24].

Visualization methods have evolved tremendously in recent years. What was once the so-called resolution limit dogma, stating that both physical parameters of the lenses and the wavelength of the light or electron beam impose a strict limit to the resolution of any microscope [46], has been overcome several times independently by the creativity of a number of inventors and scientists. Several super-resolution (SR) microscopic techniques have become available, each offering advantages and disadvantages. Electron microscopy, the high-resolution visualization method available since the 1930s, has also recently evolved tremendously, with the ability to localize molecules in a way that can be quantified, and with cryo and 3D methods, and combinations of those, readily available to the scientific community. This review targets the researchers trying to understand the molecular interactions at nanoscale resolution with a special interest in learning the latest information on the visualization methods. We give a brief explanation of modern approaches, discuss their advantages and disadvantages, and give an outlook into the future steps necessary to elucidate the mechanism of changes in inhibitory synapse plasticity due to NDs and to facilitate future drug discovery. However, this review is methodologically limited to modern visualization methods and does not include molecular biology and electrophysiological approaches. Moreover, we focus on research on inhibitory synapses in the period between 2016 and 2021.

## 2. Discovering Details of Synapses with Electron Microscopy

**Figure 2:**
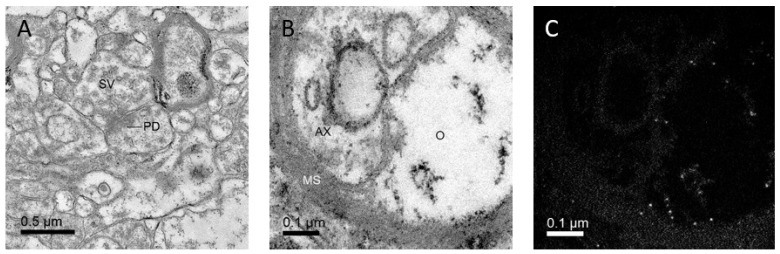
Conventional electron microscopy allows visualizing synapses and analytical electron microscopy allows visualizing iron-loaded ferritin. (**A**): Conventional electron micrograph of a synapse in a human cortex sample. Synaptic vesicles (SV), postsynaptic density (PD). (**B**,**C**): Conventional electron micrograph (**B**), and corresponding iron L-map (**C**) from a sample of the human globus pallidus. The bright spots in (**C**) correspond to ferritin particles within an oligodendrocyte (O). AX—axon; MS—myelin sheath.

Although electron microscopy provides the oldest high-resolution visualization method described here, it is not outdated. Recent developments enable localizing and quantifying proteins, visualizing structures in 3D at nanometer resolution, or even determining the structure of proteins at near-atomic resolution. Conventional transmission electron microscopy, for which tissue is fixed using aldehydes, embedded in resin, thin sectioned, and visualized under an electron beam, is the oldest available method that achieves sufficient optical resolution for visualizing synapses (reviewed in [47]). At least two cell processes (one presynaptic, at least one postsynaptic) are in contact at the chemical synaptic site. The synapses are characterized by an accumulation of vesicles that contain a neurotransmitter within the presynaptic terminal and a defined synaptic cleft between the pre and postsynaptic membrane, which exhibits an electron-dense area, termed postsynaptic density (Figure 2A).

Conventional transmission electron microscopy allows the counting of profiles of synapses on the sections, and this was recently used to show that potassium 2-(l-hydroxypentyl)-benzoate has a protective effect on synapse numbers in APP/PS1 mice [48]. Moreover, using a dissector as a counting frame on pairs of sections [49] allows unbiased quantification of the numbers of structures in a certain volume without the need for 3D reconstructions (reviewed in [50]), as has been performed for synapse numbers (e.g., [51,52,53,54,55,56,57]), or even immunolabelled cell types [58] within the period covered by this review.

Synapses in which the postsynaptic density appears thicker on electron micrographs than the presynaptic density are called Gray Type I, and synapses with an equal thickness of pre and postsynaptic thickness, Gray Type II [59]. There are many hints that Gray Type I synapses are usually excitatory, whereas Gray Type II synapses are usually inhibitory (reviewed in [47]). However, the concept that the thickness of the postsynaptic density on thin sections in EM suffices for distinguishing between excitatory and inhibitory synapses was challenged because many exceptions have been found (reviewed in [47]). A reliable marker for inhibitory synapses is the neurotransmitter receptors that are situated along the postsynaptic membrane. Even if the morphology of a synapse does not suffice to identify it as either excitatory or inhibitory, [60] there is evidence for consistent differences between GABAergic and glutamatergic synapses. Tao and co-workers [60] showed, by correlating fluorescence microscopy with cryo-electron tomography, that γ-aminobutyric acid type-A receptors (GABAAR) in primary cultures of the rat hippocampus are situated at a postsynaptic density consisting of thin sheets, whereas PSD-95, which is connected with excitatory glutamatergic receptors, exists in a thicker, mesh-like structure. This confirmed the hypothesis that different synapse types have different ultrastructural appearances.

Many neurotransmitters have either inhibitory or excitatory receptors, so if a specific receptor type can be labeled at the postsynaptic membrane, then both the specific neurotransmitter released at the synaptic site and the sign of the synapse (whether it is excitatory or inhibitory) can be made clear. Immunogold methods for transmission electron microscopy allow visualizing neurotransmitter receptors and at the same time identifying synapses. For these, either the sample or a thin section containing the sample is bathed in a specific antibody solution, and then a gold label is brought to bind close to this site by applying a gold-coupled secondary antibody or its fragment.

If chemical fixation and dehydration at room temperature is replaced by high-pressure freezing [61] and freeze substitution, the ultrastructure of the sample is preserved in a near-native state (e.g., [62]). Moreover, high-pressure freezing allows much more rapid fixation than chemical fixation. This has led to the development of flash-and-freeze technology [63,64], in which optogenetics can be combined with immediate high-pressure freezing after the tissue sample has been stimulated with light. This can give detailed 3D information of synaptic vesicle trafficking in different activation states, especially when combined with 3D methods, such as electron tomography [65,66,67,68,69].

SDS-digested freeze-fracture replica labeling (SDS-FRL, [70]) can be performed on either chemically-fixed or high-pressure frozen tissue samples. It allows studying biomembranes in combination with immunogold labeling of membrane proteins. As the antigens are exposed to the antibody, it has advantages in terms of sensitivity over pre and post-embedding immunogold techniques [71] and allows a reliable quantification [72]. Thus, this provided an elegant way of quantifying receptor numbers and was used to demonstrate that the density of metabotropic γ-aminobutyric acid type-B receptors (GABABR) are reduced in the dentate gyrus of an AD model mouse expressing mutated human amyloid precursor protein and presenilin1 (APP/PS1) [73,74,75].

Immunogold methods have revealed data that are relevant to answering the question: what induces the changes in synapses during the course of AD? Using immunogold methods performed on thin sections, in conjunction with super-resolution microscopic methods, Pickett et al., 2016, detected AβO accumulation at postsynaptic sites, although it was also found in cell bodies, dendrites, and mitochondria [18].

Several recent advances in technology now allow the use of scanning electron microscopes for studying large volumes in 3D at an electron microscope resolution (e.g., [76]; reviewed in [77]), and tilting the sections in the beam and later reconstructing the structures from its projection at different tilt angles (electron tomography) enables detailed 3D information of the subcellular structures situated within a cell or tissue section (reviewed in [78]). Electron tomography of frozen, hydrated, or freeze substituted, resin-embedded samples has begun to allow elucidation of the molecular buildup of the cytomatrix of the active zone of the presynaptic site and of the postsynaptic site [79,80,81,82], reviewed in [83,84]. Correlating immunofluorescence with SEM and electron tomography with immunogold, [85] Orlando et al. showed that synaptic potentiation induces increased GABAAR clustering in primary cultures of mouse hippocampal neurons.

Proteins can be frozen and then directly visualized in a cryo-transmission electron microscope, allowing their 3D structure to be determined at a rapidly improving resolution (reviewed in, [86]) from averaging the particles that share the same orientation. This technique has become a major tool for structural biologists and has received a boost since it led to Jaques Dubochet, Joachim Frank, and Richard Henderson being awarded the Nobel Prize in Chemistry in 2017 [87]. Cryo-EM enables the determination of the 3D structure of proteins and has the advantages that complexes, various conformations, or interaction sites with binding molecules can be determined at several nm resolutions in 3D. This will allow the studying of the binding sites of potential drugs for neurodegenerative disorders in detail. Thus, both the GABAAR [88,89,90,91]; reviewed in [92], and the GABABR [93,94,95,96], reviewed in [97], were recently described using cryo-EM in full length in different conformations. Of note, one isoform of the GABABR is found in axons as it contains an axon-sorting signal [98] that has recently been found to bind to secreted APP, reducing vesicle release when bound [99].

## 3. Super-Resolution Imaging

Synapse-associated proteins essential for neuronal transmission can be revealed by molecular and cellular neuroscience techniques. Precise roles of those proteins are dependent on their location, but synaptic structural components are highly compressed within narrow areas that cannot be resolved by conventional light microscopy due to diffraction limits (approximately 200 nm for conventional light microscopy) [100]. New SR methods enable the diffraction limit of light microscopy to be overcome. Here, we present recent imaging improvements due to structured illumination microscopy (SIM), stimulated emission depletion microscopy (STED), and photoactivated localization microscopy (PALM) or stochastic optical reconstruction microscopy (STORM).

To achieve a super-resolved SIM image, the sample is illuminated with a series of excitation light patterns and the image is reconstructed out of the interference moiré patterns of the structured illumination and underlying labeled structure. This method is suitable for live-cell imaging because it uses low laser power and high frame rates. The use of typical fluorescent dyes and fluorescent proteins (up to four different colors) makes it very convenient. However, it has a lower resolution than other SR methods [101,102].

Recently, Schürmann and colleagues [103] used SIM to study the late-onset Alzheimer’s disease risk factor BIN1 and showed that this protein is abundant in postsynaptic compartments, including dendritic spines. In order to image key components of the inhibitory postsynaptic domain and presynaptic terminal, 3D SIM was used in combination with STED. Cosby and colleagues [104] revealed that inhibitory synapses are organized into nanoscale sub-synaptic domains (SSDs) and that, in response to elevated activity, synapse growth is mediated by an increase in the number of postsynaptic SSDs. Notably, they found no difference in the number of SSDs per synaptic compartment, as identified by STED and SIM, indicating that SIM provides sufficient resolution to identify inhibitory SSDs.

The STED microscope [105] works with two lasers and is basically built up as a confocal laser scanning microscope: one regular laser is used to excite the sample and the other one as a doughnut-shaped depletion laser. The role of the doughnut depletion laser is to deactivate fluorophores selectively by forcing them to emit photons in a higher wavelength, thus minimizing the effective area of illumination to a smaller focal point. The method achieves a high resolution of <40 nm, making it the method of choice for correlative microscopy. Dependent on the scanning area, high frame rates can be achieved, although full-frame imaging is rather slow. The method cannot be used on living cells because its laser power is too high [106].

Yu and coauthors [107] used STED to clarify the pre and postsynaptic subcellular locations of fragments involved in the amyloidogenic pathway in primary neurons with a focus on 42 amino acid-long amyloid-beta peptide (Aβ42) and its immediate substrate AβPP C-terminal fragment (APP-CTF).

De Rossi and colleagues [108] investigated the role of BIN1 (late-onset Alzheimer disease risk factor) function in the brain using conditional knockout (cKO) models. dSTORM and immuno-EM were used to elucidate BIN1 location, predominantly at presynaptic sites in glutamatergic synapses. Confocal and STED microscope analysis of presynaptic morphology in cKO mice revealed a decrease in the density of presynaptic sites and the size of presynaptic protein clusters.

Aβ42 is a peptide, which forms neurotoxic oligomers and amyloid plaques and plays a key role in the loss of synapses in AD. STED and dSTORM in combination were used to show that Aβ42 is present in small vesicles in presynaptic compartments but not in postsynaptic compartments in the neurites of hippocampal neurons [109].

STORM exploits the photo-switchable nature of specific fluorophores for temporal separation of fluorescent signals, which otherwise would overlap spatially. In each imaging cycle, only a fraction of fluorophores are turned on, and fluorophore positions are obtained by fitting a PSF or Gauss distribution from a series of imaging cycles. The *x*/*y* positions are used to reconstruct a super-resolved image with a resolution <20 nm. STORM can reach molecular-scale resolution; however, special dyes and protocols are required, and imaging times can exceed more than 10 min per image [110].

dSTORM was used by Nanguneri et al. [111] to obtain nanoscale images. These images were analyzed with especially adopted supervised learning methods to understand the heterogeneity in the organization of F-actin in dendritic spines of primary neuronal cultures from rodents. The results were validated using ultrastructural data obtained from platinum replica electron microscopy [112].

Dual-color direct STORM was used to image glycine receptors (GlyRs) and GABAARs at mixed inhibitory synapses in spinal cord neurons to examine how different inhibitory receptors are regulated. This study revealed that SSDs are aligned in trans-synaptic nanocolumns at inhibitory synapses while being differentially spatially organized at mixed inhibitory synapses [113].

A powerful combination of STORM and STED microscopy was used to show that γ-secretase is present in both the pre and postsynaptic compartments. This enzyme is enriched very close to the synaptic cleft in the postsynaptic membrane, as well as to NMDA receptors, demonstrating that γ-secretase is present in the postsynaptic plasma membrane. A correlation between γ-secretase activity and synapse maturation was suggested [111].

Paasila et al. [114] visualized the interactions between presynaptic terminals and microglia in situ, using dSTORM. The procedure they described opens the spectrum of molecular imaging using antibodies and SR microscopy to the analysis of routine formalin-fixed paraffin sections of the archival human brain. Especially interesting is the investigation of microglia-synapse interactions in dementia [115].

A newly developed combination of STED and STORM (MINFLUX, Abberior, company founded by Stefan Hell, https://abberior-instruments.com/knowledge/publications/, accessed on 17 November 2021) combines single-molecule activation with imaging using a super-fast STED-microscope to triangulate the exact position of the fluorophore. These results are in very high resolution (2 nm) and the imaging speed is faster compared with STORM [114,116].

PALM works on a similar principle as STORM, but unlike STORM, which is used for fixed cells, PALM is used to image living, transfected cells. Particle tracking can be performed on this device as well (sptPALM) [117].

SEQUIN (Synaptic Evaluation and Quantification by Imaging Nanostructure) is a new method that combines special tissue processing with Airyscan image scanning microscopy and special automated software. First, the tissue is immunolabeled with fluorescent antibodies for pre and postsynaptic sites, then it is cleared and finally put under the microscope. Automated software is used for image processing and functional synapse identification by matching closely apposed pre and postsynaptic markers. The resolution is 140 nm, and 97% of synapses are identified. In a study of a murine model of AD by Sauerbeck et al. [118], synaptopathic alterations were revealed in the vicinity of amyloid plaques. Sequin revealed a proximity-dependent loss of synapses in such regions [118].

SR methods are an option when higher resolution is needed than can be obtained using confocal microscopy. Although EM provides the highest resolution and immunogold for EM has the advantage that unlabeled structures are visible in addition to gold-labeled ones, there are limitations in labeling density, as well as in the number of proteins that can be labeled and imaged simultaneously. Immuno-gold particles are distinguished from each other by their size, and there has to be a noticeable size difference in gold particles used to label different proteins of interest. Other limitations are that living cells or tissues cannot be imaged with conventional EM and that 3D-EM methods are time-consuming. The high resolution provided by EM may not be necessary to answer a particular question.

**Figure 3:**
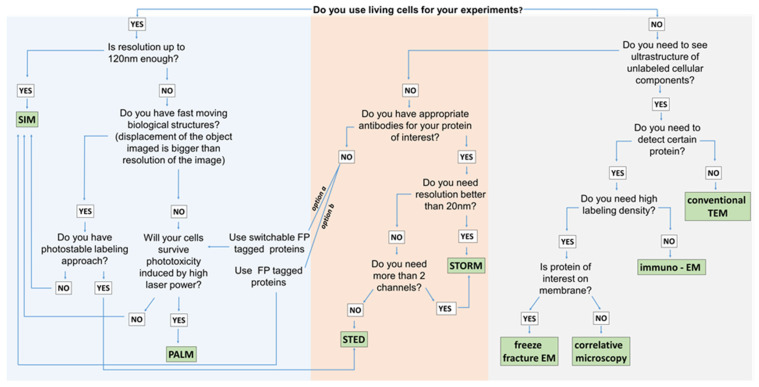
Guidelines for choosing appropriate visualization methods (SIM—structured illumination microscopy; PALM—photo-activated localization microscopy; STED—stimulated emission depletion microscopy; STORM—stochastic optical reconstruction microscopy; EM—electron microscopy; TEM—transmission electron microscopy).

How do we choose a particular super-resolution method? A compromise must be made between resolution, speed, and the number of available color channels. For example, if living cells are imaged, a method with low laser power is required. If there is no need for a resolution higher than 60 nm and there are more than two proteins to visualize, SIM would be the best choice. Otherwise, if the aim is to visualize only two proteins with very high resolution, and cells can be fixed, the method of choice would be STED (see Table 2 and Figure 3).

## 4. How to Visualize Iron in Neurodegenerative Disorders

The connection between iron and neurodegenerative diseases has been extensively reviewed during the survey period for this review [119,120,121]. Iron deposits are found in the brain of people suffering from ND [122,123,124,125], overlapping with both amyloid deposits and tau neurofibrillary tangles [125]. Excessive iron load is a major contributor to AD, but it is still unclear if it is a cause [121]. The action of iron in AD is threefold. First, iron is known to be able to upregulate APP transcription and aggregation [126,127]. Because APP has been shown to increase iron export from neurons [128], there is potentially a vicious circle of ever-increasing APP and iron accumulation. Second, the presence of Ab oligomers has been shown to reduce ferritin iron chemically [129], increasing the amount of iron in a ferrous form that can cause radical production in the cell and thus damage the cell and its compartments (e.g., [130]). Third, recent findings indicate ferroptosis—a necrotic process involving iron and lipid oxidation—is responsible for the neuron loss that occurs during Alzheimer’s disease [131].

Visualization methods of iron accumulation exist both at low and high resolution. At low resolution, Magnetic Resonance Imaging (MRI) allows not only to visualize plaques [26] but also iron mapping [26,122,132,133]. At electron microscope resolutions, analytical electron microscopy allows localizing chemical elements within the samples.(Figure 2B,C; [125,134]). Similarly, X-ray spectromicroscopy can show the chemical elements and their oxidation state within the sample [129].

## 5. Discussion and Conclusions

Microscopy is an extremely rapidly advancing field, and, as we have shown, several of the newly available techniques have already been applied for studying synaptic plasticity in Alzheimer’s disease. In the past, microscopists were limited by two perceived physical limits in microscopy: first, the diffraction limit [46], which stated that the wavelength of the light or electron beam, along with physical parameters of the lenses, sets a limit to the resolution of any microscopes. The resolution of conventional light microscopes is limited to approximately 200 nm. This physical limitation has been overcome with the invention of super-resolution microscopes, which work around it in several different ways and offer spatial resolutions similar to that of electron microscopes [100]. The second limit is that high spatial resolution can only be achieved at a low temporal resolution and vice versa, meaning live processes can only be visualized at a low spatial resolution, and only dead tissue or cells can be used for high-resolution studies [15]. As we have shown here, this second limit still requires some compromise in terms of spatial resolution if the high temporal resolution is required and vice versa (Figure 3) but is also becoming resolved with the availability of super-resolution microscopes for live cells (Figure 3).

The visualization method of choice depends on the specific questions to answer. As Figure 3 and Table 2 show, there are a wealth of options. If the tissue or cells are dead (fixed and embedded) and ultrastructural features of the compartments are important, EM will be the method of choice, and if proteins need to be labeled, super-resolution methods, such as STORM or STED, can be applied as an alternative to immunogold methods for EM.

Furthermore, iron, which plays a role in neurodegenerative disorders (reviewed in [119,121,135]), can be directly visualized using analytical microscopy (Figure 2B,C). Last but not least, optogenetics can be combined with modern visualization methods, such as super-resolution microscopy or high-pressure freezing and EM [63,64]. This will enable dissecting major steps in the etiology of neurodegenerative disorders.

In summary, microscopy has recently advanced tremendously, and major improvements can be expected in the research of neurodegenerative disorders if new visualization methods are used.

## 6. Materials and Methods

For EM iron visualization, tissue was taken from deceased humans from a routine autopsy. After the pathologist had released the tissue, it was embedded in resin, thin sectioned, contrasted using lead citrate and platinum blue, and visualized using a Tecnai G2 transmission electron microscope operated at 200 kV. The iron L elemental map was made with a Gatan Quantum GIF energy filter, using the settings suggested by Gatan. All the figures were created with BioRender.com.

## Figures and Tables

**Table 1:** The most common neurodegenerative diseases and their specific effect on inhibitory synapses (IS). The current treatment options and drugs mentioned here are used only to alleviate the symptoms in order to halt the progression of the disease.

Type	Profile	Major Symptoms	Impact on IS	Treatment	Drug Target Site	Reference
Alzheimer’s disease	MRI AβO hTau NFT	Dementia cognitive impairment	Loss of GABAergic neurons	Memantine	NMDAR antagonist—postsynapse of EN	[6,7,20,22,25,26]
Parkinson’s disease	MRI α—Synuclein Lewy neurites Lewy bodies	Dementia Bradykinesia Rigidity Rest tremors	Loss of dopaminergic neurons	Levodopa combined with dopamine agonists	Presynaptic nerve terminals	[27,28,29,30,31,32]
Multiple Sclerosis	MRI scarring of tissue demyelination oligoclonal bands Neurofilaments	cognitive impairment defects in vision muscle spasms fatigue	loss of motor neurons loss of selective inhibitory neurons	Immunosuppressants Cytokines	Myelin sheath Axon fibers	[27,33,34,35,36,37]
Amyotrophic Lateral Sclerosis	Neurofilaments	cognitive impairment frontotemporal dementia muscle spasms and atrophy	loss of inhibitory cortical interneurons	Riluzole Baclofen	blocks NMDAR–postsynapse inhibits glutamate release—pre-synapse GABABR agonist–postsynapse	[28,29,30,31,32,38,39,40]
Huntington’s disease	MRI mHTT protein Neurofilament light protein	cognitive impairment dementia chorea	loss of GABAAR	Tetrabenazine Antipsychotics	inhibits VMAT-2—presynapse	[41,42,43,44,45]

Abbreviations: EN—excitatory neurons; IN—inhibitory neurons; MRI—Magnetic Resonance Imaging; NFT—Neurofibrillary tangles, hTau—hyperphosphorylated tau; GABA—Gamma Amino Butyric Acid; NMDAR—N-methyl D-aspartate Receptors; GABAAR—γ-aminobutyric acid type-A receptors; GABABR—γ-aminobutyric acid type-B receptors; mHTT—mutant Huntington’s protein; VMAT—vesicular monoamine transporter.

**Table 2:** Comparison of technical performances of most commonly used super-resolution methods.

	SIM	STED	PALM/STORM
Resolution	x/y = 60–140 nm z = 120–250 nm	x/y = 2–40 nm z > 4300 nm MINFLUX has 2 nm resolution; others have resolution of 20–80 nm; very much dependent on the device	x/y = 1–40 nm z = 20–50 nm strongly dependent on chemical method of on/off switching
Live imaging	240 fr/s Lattice SIM, Zeiss	10–20 fr/s depending on area	0.2 fr/s
Laser power	1–10 W/cm^2^	100 MW/cm^2^	1–25 kW/cm^2^
Colours	4	Max 2	2–4
Dyes	typical fluorescent dyes	Atto647N, Chromeo 494	AF647, mEos2
